# Effect of microwave radiation on adult neurogenesis and behavior of prenatally exposed rats

**DOI:** 10.1016/j.ibneur.2024.08.007

**Published:** 2024-08-27

**Authors:** Alexandra Popovičová, Enikő Račeková, Marcela Martončíková, Kamila Fabianová, Adam Raček, Monika Žideková

**Affiliations:** Institute of Neurobiology, Biomedical Research Center, Slovak Academy of Sciences, Šoltésovej 4, Košice 040 01, Slovakia

**Keywords:** Rostral migratory stream, Dentate gyrus, Prenatal irradiation, Postnatal neurogenesis, Microwave radiation

## Abstract

Postnatal neurogenesis appears to be highly sensitive to environmental factors, including microwave electromagnetic radiation (MWR). Here, we investigated the impact of MWR during intrauterine development on juvenile and adult neurogenesis in the rostral migratory stream (RMS) and the dentate gyrus of the hippocampus in the rat brain, as well as its effect on animal behavior. Female rats were exposed to MWR at a frequency of 2.45 GHz for 2 hours daily throughout pregnancy. The offspring of irradiated mothers survived to either juvenile age or adulthood. The brains of the rats were subjected to morphological analysis, assessing cell proliferation and death in both neurogenic regions. In the RMS, the differentiation of nitrergic neurons was also investigated. The effect of MWR on behavior was evaluated in rats surviving to adulthood. Prenatal MWR exposure caused significant changes in the number of proliferating and dying cells, depending on the age of the animals and the observed neurogenic region. In addition, MWR attenuated the maturation of nitrergic neurons in the RMS in both juvenile and adult rats. Morphological alterations in neurogenesis were accompanied by changes in animals’ behavior. Affected neurogenesis and changes in animal behavior suggest a high sensitivity of the developing brain to MWR.

## 1 Introduction

The continuous generation of new neurons in the adult mammalian brain takes place in two specific regions, in the subventricular zone (SVZ) of the lateral ventricles and in the subgranular zone (SGZ) of the dentate gyrus (DG) of the hippocampus (Doetsch et al., 1997, Imayoshi et al., 2009). Importantly, increasing evidence confirms that neurogenesis persists in the adult human brain as well (Curtis et al., 2012, Bergmann et al., 2015, Boldrini et al., 2018).

Stem cells present in the SVZ differentiate into neuroblasts that migrate over a relatively long distance through the rostral migratory stream (RMS) to the olfactory bulb (OB), where they differentiate into functional interneurons (Doetsch et al., 1997, Ponti et al., 2013). Neuroblasts gradually formed from SGZ stem cells differentiate into granular cells and integrate into existing hippocampal circuitries (Kemperman et al. 2015).

Although the process of neurogenesis in these two neurogenic areas has its specificities, in both the SVZ/RMS/OB and hippocampus, several well-characterized stages of neurogenic process have been described, from the proliferation of progenitor cells and migration of neuroblasts to neuronal differentiation, maturation and functional integration. Specific stages of adult neurogenesis in mammals have been found to be regulated by different molecular players. Among others, a free-radical signaling molecule nitric oxide (NO) has also been shown to participate in the regulation of adult neurogenesis (Gray and Cheung, 2014). The regulatory action of NO was clearly demonstrated in the RMS (Packer et al. 2003; Gutièrrez-Mecinas et al., 2007). Previously, we have identified mature NO-producing neurons located directly in the RMS of adult rats (Blaško et al. 2013). We have also shown that nitrergic neurons appear in the RMS after the first postnatal week and then their development continues until the end of the first postnatal month when they reach the morphology seen in adult animals (Račeková, 2003).

Studies in rodents have revealed important functional roles of newly generated neurons in both neurogenic regions. It has been found that new OB interneurons are necessary for proper olfactory processing and behavior (Takahashi et al., 2018), and adult hippocampal neurogenesis is functionally linked to learning and memory and emotional processing (Deng et al. 2010). In addition to the physiological role of the postnatal neurogenesis, its contribution to pathological processes in the mammalian brain has also been described. For example, SVZ and SGZ progenitor cells can be activated in pathological conditions such as traumatic or ischemic brain injury (Dash et al., 2001, Faiz et al., 2015). Specific alterations of postnatal neurogenesis may also be related to the development of some neurodegenerative and psychiatric diseases (Winner et al. 2011). Furthermore, adult neurogenesis has been shown to be modulated by various environmental factors.

One of the most serious exogenous factors with a potential adverse effect on living organisms, including humans, appears to be non-ionizing electromagnetic radiation (EMR). Non-ionizing EMR includes microwave radiation (MWR) with frequencies ranging from 300 MHz to 300 GHz, which are the frequencies on which mobile phones and wireless technologies such as Wi-Fi routers, GPS, portable Bluetooth gadgets and many others operate. For example, the most widely used frequencies for Wi-Fi are 2.45 and 5 GHz (Zhang et al. 2015). Excessive use of these electronic devices results in dramatically increased pollution of our environment by MWR. Investigations on experimental animals have shown that increased exposure to MWR may lead to hazardous effects on nervous system function, brain activity and cognitive behavior (Dong et al., 2011, Zhao et al., 2012), and the brain has been recognized as the most vulnerable organ to this type of radiation (Mumtaz et al., 2022, Hao et al., 2015, Zhi et al., 2017). Among brain structures, areas of the brain where new neurons are generated even after birth appear to be highly sensitive to radiation (Raček et al., 2018, Lin et al., 2021).

In our previous studies, we showed an adverse effect of MWR on neurogenesis in the olfactory neurogenic region of rats exposed to MWR in adulthood (Orendáčová et al. 2009) or in juvenile age (Raček et al. 2018). The aim of the present work was to investigate proliferation and cell death in the RMS and in the DG of the hippocampus of rats after their exposure to MWR during intrauterine development. Morphological and quantitative analyzes were performed at two survival times after irradiation, in juvenile and adult animals. Given the important role of NO in the regulation of neurogenesis, we monitored the maturation of nitrergic neurons within the RMS after both survival times. We also aimed to observe the effect of MWR on the behavior of prenatally irradiated animals that survived to adulthood.

## 2 Material and methods

### 2.1 Animals

Three-month-old, female Wistar albino rats (n=6) were included in the study. Handling and all experimental procedures were performed in accordance with the approval of the Animal Care Ethical Committee of the Institute of Neurobiology of the Biomedical Research Centre, Slovak Academy of Sciences and the State Veterinary and Food Administration of the Slovak Republic. The animals were kept in standard conditions with 12/12 h light/dark cycle and with free access to food and water. The females were mated with a three-month-old male rat (n=1) of the same strain. Vaginal smears were examined under a light microscope and the presence of spermatozoa was considered as the first day of pregnancy. The pregnant rats were then divided into control and experimental groups (3 rats/group) and placed in a separate cage.

### 2.2 Experimental intervention

Pregnant rats of the experimental group (n=3) were whole-body exposed to a pulsed-wave microwave radiation at the frequency of 2.45 GHz and mean power density of 2.8 mW/cm^2^ at an average specific absorption rate (SAR) of 1.73 W/kg in a purpose-designed exposure chamber. Power density measurements inside the exposure chamber were performed with a portable electromagnetic field meter (LUTRON EMF-819, Taiwan). The rats were irradiated for 2 hours per day throughout their pregnancy. To eliminate additional stressful stimuli during exposure, the animals were left in their home cage with access to water and food ad libitum. Pregnant rats of the control group (n=3) were placed in the exposure chamber for an equivalent amount of time, but were not exposed to radiation. The offspring of irradiated as well as control mothers survived either to juvenile age (5 weeks; irradiated: n=12, control: n=12) or to adulthood (3 months; irradiated: n=12, control: n=12).

### 2.3 Behavioral testing

The effect of MWR on animal behavior was evaluated in a group of rats of both sexes (the ratio of males to females was the same in the experimental and control groups) that survived to adulthood. Three-month-old rats irradiated during intrauterine development (n=12) and sham-exposed control rats of the same age (n= 12) were tested in the open field test, elevated plus maze and dark/light box test.

In order to evaluate the locomotor activity and the anxiety-related behavior in rats, we employed the open field test, as described previously (Fabianová et al., 2014, Kraeuter et al., 2019b, Kraeuter et al., 2019a). The apparatus had the shape of a rectangle with a size of 61 ×45 x 33 cm, and the walls of the open field apparatus were made of plexiglass. Each animal was tested individually. The rat was placed in the middle of the open field and tested for 8 minutes. During testing, the experimenter remained outside the testing room. Time spent in the central zone (s), total distance traveled (cm), and average movement speed (cm/s) were measured using EthoVision XT, version 7.0. Subsequent manual video analysis performed by an experimenter blind to group assignment scored: grooming (s), rearing (number), the number of crossings of the middle point of open field, the time spent by the walls of open field (s), and defecation (number). After each test, the apparatus was cleaned with ethanol.

To test exploratory behavior and anxiety in rats, we used an elevated plus maze (Kraeuter et al., 2019b, Walf and Frye, 2007). The elevated plus maze consisted of four elevated arms which radiate from a central platform, forming a plus shape. The two opposite arms had closed walls (except the ceiling, entrance, and exit points), and the remaining two opposite arms were open. The elevated plus maze was located at a height of about 0.5 m above the ground due to limited contact with the floor. We tested the rats individually. The rat was placed on the central area of apparatus and tested for 5 minutes. During testing, the experimenter remained outside the testing room. The time spent in the open/closed arms (s) was captured by a camera and evaluated by EthoVision XT software, version 7.0. Subsequent manual video analysis performed by the experimenter included the center crossings (number), rearing (number), grooming (s), time spent in open arms (s) and defecation (number).

The dark/light box test is the most commonly used test to evaluate locomotor activity, anxiety, and emotional reactivity in rodents (Arrant et al., 2013, Raček et al., 2018). The box was divided by a wall with a hole into light and dark part. We placed the rat inside the dark box and tested it for 5 minutes. During the testing period, the experimenter stayed outside the testing room. Time spent in the dark box (s), time spent in the light box (s), total distance traveled in the open area (cm), average movement speed (cm/s) were evaluated by EthoVision XT software, version 7.0. Defecation, number of entries in the light box, the latency to the first poke into the light part (s), the time spent with head poked into the light part (s), and the number of pokes into the light part were scored manually.

### 2.4 Histological procedures

Both juvenile and adult prenatally irradiated rats as well as age-matched control rats were anesthetized with isoflurane anesthesia and received intraperitoneal administration of chloral hydrate (300 μl/100 g animal weight). Under deep anesthesia, the rats were transcardially perfused through the left ventricle with saline, followed by 4 % paraformaldehyde in 0.1 M phosphate buffer (PBS). After perfusion, the rats were decapitated, and the brains were removed from the skulls. The brains were then postfixed in 4 % paraformaldehyde at 4°C. The next day, the brains were transferred into cryoprotective solution - 30 % sucrose for 48 hours. Subsequently, 30 μm sagittal and coronal sections were cut on the cryostat to examine the RMS and the hippocampus, respectively (n=6 for each method of processing).

#### 2.4.1 Ki-67 immunohistochemical analysis

Sections were washed in 0.1 M PBS solution 3 times for 10 minutes each. To suppress peroxidase activity, the sections were immersed in 3 % H_2_O_2_ solution for 5 minutes and washed again in PBS. Blocking of non-specific protein binding was achieved by using 5 % NGST (normal goat serum with 0.3 % Triton in 0.1 PBS) for 2 hours. The sections were then incubated overnight (18 hours) with rabbit anti Ki-67 monoclonal antibody (1:1000, Abcam, Cambridge, UK) dissolved in 1 % NGST. The following day, the sections were washed again in PBS and incubated for 2 hours with biotinylated goat anti-rabbit secondary antibody (Vector, CA, USA), diluted 1:200 in 1 % NGST. Subsequently, the sections were washed in 0.1 M PBS, submerged in avidin-biotin complex (ABC, Vector, CA, USA) for 1 hour, washed in PBS, and treated with diaminobenzidine solution (1:9, Roche Diagnostics, Germany). Finally, the sections were mounted on slides, dried, and coverslipped with Entellan (Merck, Germany).

#### 2.4.2 NADPH-diaphorase histochemistry

Free-floating sections were incubated in 0.1 M PB, pH 7.4, containing 0.4 mg/ml of Nitroblue tetrazolium, 0.8 mg/ml of NADPH (Sigma), 0.3 % Triton X-100 dissolved in 0.1 M PB (pH 7.4), 5 mg/ml malic acid, and 4 mg/ml magnesium chloride for 1 h at 37ºC. The sections were then rinsed in 0.1 M PB, mounted on gelatin slides, air-dried overnight, cleared with xylene, and cover-slipped with Entellan.

#### 2.4.3 Fluoro - Jade C histochemical analysis

First, sections mounted on slides were placed in a thermostat for 30 minutes (50–55°C). Subsequently, the slides were immersed in absolute alcohol for 3 minutes, followed by a 2-minute immersion in 70 % alcohol and 1 minute in distilled water. To suppress background staining, the sections were treated with 0.06 % KMnO_4_ for 13 minutes and then rinsed with distilled water for 1 minute. Following this, the sections were stained in FluoroJade-C (FJ-C) solution for 90 minutes in the dark. To prepare a 0.01 % stock solution of FJ-C), 10 mg of FJ-C was dissolved in 100 ml of distilled water. The staining solution (0.001 %) was prepared before staining using 1 ml of FJ-C stock solution and 99 ml of 0.1 % acetic acid. Finally, the slides were washed 3 times with distilled water (1 minute each), were dried in the dark, and mounted with Fluoromount.

### 2.5 Quantitative analysis in the RMS

For the analysis of proliferative activity in the RMS, only those sections on which the entire RMS was visible (7–10 sections per animal) were used. The caudal limit of the RMS was defined as the point where the lumen of the lateral ventricle opens up, and the rostral limit was defined as the point where the OB begins. Sagittal sections were observed and scanned under a 40x oil immersion objective using a digital camera (DP50) mounted on a microscope (Olympus BX51). Ki-67+ cells were counted using Disector 2.0 software (Tomori et al., 2001) in three parts of the RMS (the vertical arm, the elbow, and the horizontal arm), and the average of the values in each of the three parts was calculated. The number of proliferating cells in the RMS was expressed as the mean number of Ki-67+ cells per 1 mm^3^.

For the analysis of NO-producing neurons within the migratory pathway, photographs of the sagittal sections were taken using a Slide Scanner Aperio AT2 microscope (Leica) at 20x magnification. Individual NADPH-d+ cells were manually counted using Image J 1.8.0_112 software in all sections where the entire RMS was visible. The results were expressed as the mean number of nitrergic neurons in the RMS per section.

Dying cells in the RMS were quantified in images of individual sections, taken using a fluorescence microscope (Olympus BX-51 fitted with an Olympus DP71 digital camera system) at 20x magnification. FJ-C+ cells were counted manually using Image J 1.8.0_112 software on the sections showing the entire RMS (7–10 sections per the animal). The results were expressed as mean number of FJ-C+ cells in the RMS per section.

### 2.6 Quantitative analysis in the hippocampus

Proliferative activity in the hippocampus was analyzed on serial coronal sections (16 sections/animal). The sections were examined, and images were captured by a Slide Scanner Aperio AT2 microscope (Leica) at 20x magnification. Ki-67+ cells in the dentate gyrus of the hippocampus were counted manually using Image J 1.8.0_112 software. The number of labeled cells was expressed as number of cells per section.

To quantify dying cells in the hippocampus, serial coronal sections (16 sections/animal) were examined, and images were captured under a fluorescence microscope (Olympus BX-51 fitted with an Olympus DP50 digital camera system) at 20x magnification. Cells were counted manually with supporting Image Tool software (Image J 1.8.0_112). The results were expressed as mean number of FJ-C+ cells in the hippocampal gyrus dentatus per section.

### 2.7 Statistical analysis

The data were analyzed using GraphPad Prism 6.0 software. An unpaired t-test was used to perform the statistical analyses. All data are presented as mean ± SEM. We considered the values to be significant at p<0.05 (*), p<0.01 (**), p<0.001 (***), p<0.0001 (****).

## 3 Results

In this study, we examined the effect of MWR on neurogenesis in juvenile and adult offspring whose mothers were irradiated during pregnancy. We assessed cell proliferation, differentiation of nitrergic neurons, and cell death in two neurogenic areas: the RMS and the hippocampal DG.

### 3.1 Morphological changes in the RMS

#### 3.1.1 Cell proliferation

**Fig. 1:**
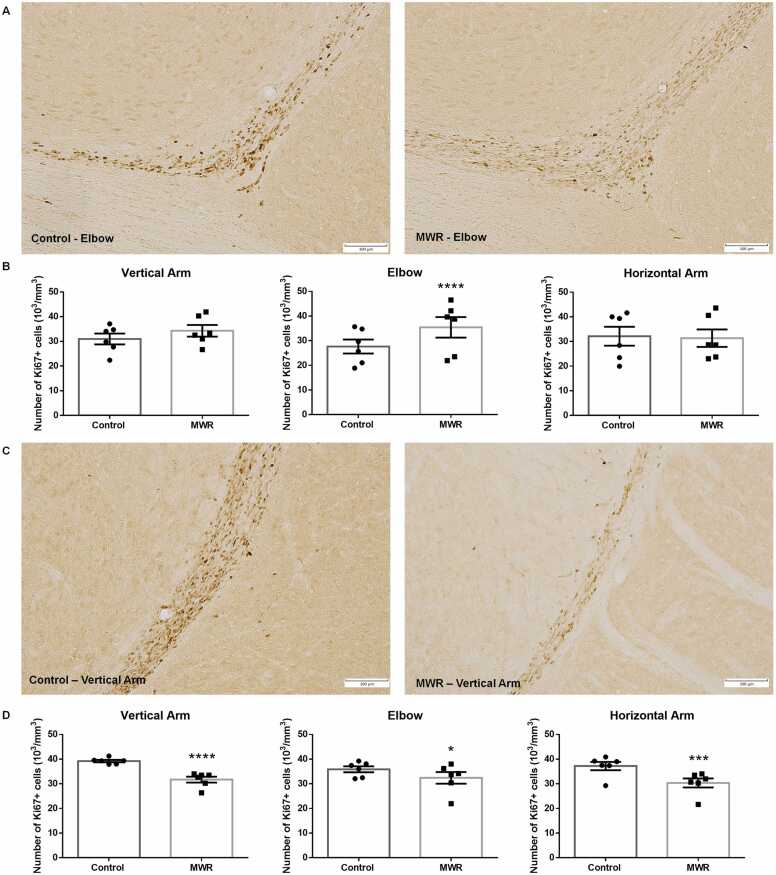
Proliferating cells in the RMS of control and prenatally irradiated rats. (A) Representative micrographs showing Ki-67+ cells in the elbow of the RMS of control and prenatally irradiated juvenile rats (B) Quantification of Ki-67+ cells in individual parts of the RMS of juvenile control and prenatally irradiated rats (C) Representative micrographs showing Ki-67+ cells in the vertical arm of the RMS of control and prenatally irradiated adult rats. (D) Quantification of proliferating Ki-67+ cells in individual parts of the RMS of adult rats. MWR - microwave electromagnetic radiation. Scale bar 100 µm. Mean ± SEM. Statistical significance *p <0.05, *** p <0.001, **** p <0.0001.

Exposure of rats to MWR during intrauterine development caused noticeable changes in the density of proliferating cells in the RMS of juvenile rats (Fig. 1A). Quantitative analysis revealed an increase in the number of Ki-67+ cells in the RMS of prenatally irradiated juvenile rats compared to age-matched control animals, which was highly significant in the elbow part of the RMS (control: 27.63 ± 1.16 SEM, MWR: 35.46 ± 1.48 SEM, p < 0.0001) (Fig. 1B).

Changes in cell proliferation caused by prenatal irradiation of rats persisted into adulthood. Microscopically visible alterations in the density of proliferating cells were observed along the entire RMS of 3-month-old animals, with the most prominent changes in the vertical arm (Fig. 1C). Subsequent quantitative analysis of Ki-67+ cells showed that, contrary to the observed effect in juvenile rats, the impact of MWR on cell proliferation in adult rats was characterized by a significantly lower number of dividing cells in the vertical arm (31.71 ± 1.045 SEM), elbow (32.24 ± 1.280 SEM), and horizontal arm (30.42 ± 1.323 SEM) compared to the RMS of control adult rats (vertical arm: 39.24 ± 0.800, elbow: 35.99 ± 1.014 SEM, horizontal arm: 37.25 ± 1.260 SEM; Fig. 1D).

The decrease in the number of Ki-67 positive cells was most prominent in the caudal part of the RMS – the vertical arm (Fig. 1D), characterized by the highest number of dividing cells under physiological conditions.

#### 3.1.2 Cell differentiation

Prenatal exposure to MWR strikingly affected the postnatal maturation of nitrergic neurons within the RMS. The postnatal development of NO-producing cells was incomplete; these cells retained immature neuronal features at both survival times in juvenile and adult rats.

**Fig. 2:**
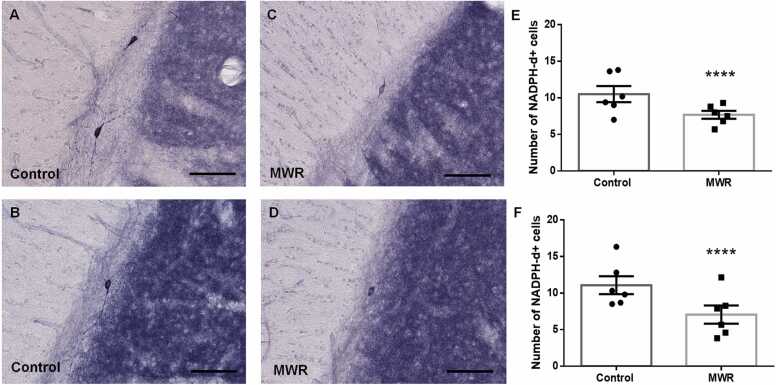
Representative micrographs of NADPH-d+ nitrergic neurons in the RMS of control juvenile (A) and adult rats (B) and MWR exposed juvenile (C) and adults rats (D). The labeled cells in control animals are multipolar with well-developed varicose processes (A, B). Nitrergic neurons in the RMS of irradiated rats have bipolar, spindle-shaped cell body with short fibers (C, D). Scale bar 200 µm. Quantification of the NADPH-d+ neurons in the RMS of juvenile (E) and adult (F) rats showed significant decrease in their number in animals exposed to MWR prenatally. Mean ± SEM. Statistical significance **p <0.01, **** p <0.0001. MWR - microwave electromagnetic radiation.

While nitrergic neurons of juvenile and adult control rats displayed the typical morphological characteristics of mature neurons with well-developed varicose processes extending deeply into the surrounding brain structures (Fig. 2A, B), nitrergic neurons in the RMS of age-matched irradiated rats had bipolar, spindle shaped body with short processes without varicosity, resembling immature neurons (Fig. 2C, D). In addition, quantitative analyses showed that the number of NO-producing cells in the RMS of both juvenile and adult rats prenatally exposed to MWR was significantly lower than in age-matched control animals. Compared to control values, the decrease was more pronounced in adult animals (juvenile: 7.91±0.44 SEM vs. 9.97±0.45 SEM, p=0.0016; adult: 7.06 ± 0.58 SEM vs. 11.05 ± 0.58 SEM, p<0.0001) (Fig. 2E, F).

#### 3.1.3 Cell death

**Fig. 3:**
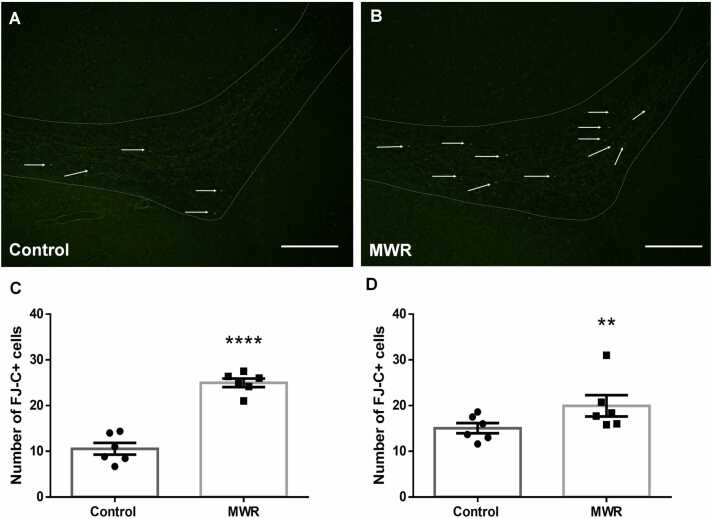
Cell death in the RMS of control and prenatally irradiated rats. Representative micrographs showing FJ-C+ cells (arrows) in the elbow of the RMS of control (A) and prenatally irradiated juvenile (B) rats. The RMS is outlined by dotted line. Scale bar 100 µm. Quantification of dying, FJ-C+ cells in the RMS of juvenile (A) (C) and adult (B) (D) rats. MWR exposure of rats during intrauterine development caused a significant increase in the number of dying cells in the RMS in both juvenile and adult rats. Results are expressed as mean ± SEM. Statistical significance ** p <0.01, **** p <0.0001. MWR - microwave electromagnetic radiation.

We investigated the effect of MWR also on cell death within the migratory pathway. Although dying FJ-C+ cells were present in the RMS both of control and prenatally irradiated juvenile rats, in irradiated animals, the density of FJ-C+ cells was visibly higher (Fig. 3A, B). Quantitative analysis confirmed that prenatal exposure to MWR caused significant increase in the number of dying cells in the whole extent of the RMS of juvenile rats (24.95 ± 1.92 SEM) compared to control animals of the same age (10.52 ± 0.55 SEM, p<0.0001) (Fig. 3A C).

The effect of MWR on cell death in the RMS was also found in adult animals irradiated prenatally. Based on fluorescent microscopic analysis, we observed an increased density of dying cells in the irradiated group compared to the control group. Quantitative analysis of FJ-C positive cells was consistent with the morphological observations. The number of dying cells was significantly higher in adult rats irradiated prenatally (19.92 ± 1.21 SEM, p=0.0037) in comparison with adult control rats (15.03 ± 1.05 SEM, Fig. 3B D).

### 3.2 Morphological changes in the hippocampus

#### 3.2.1 Cell proliferation

**Fig. 4:**
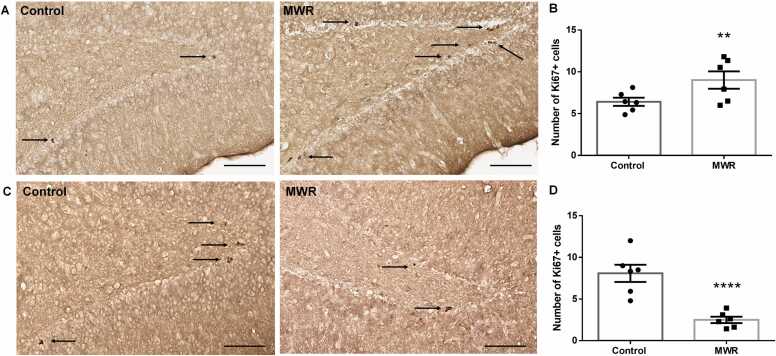
Proliferating cells in the DG of the hippocampus in control and prenatally irradiated animals. (A) Representative micrographs showing Ki-67+ cells (arrows) in the hippocampal DG of control and prenatally irradiated juvenile rats. (B) Quantification of proliferating cells in the DG of the hippocampus in juvenile rats showing significantly higher number of Ki-67+ cells in group of prenatally irradiated rats compared to that in control rats of the same age. (C) Representative micrographs showing Ki-67+ cells (arrows) in the hippocampal DG of control and prenatally irradiated adult rats. (D) Quantification of proliferating cells in the DG of the hippocampus in adult rats showing highly significant decrease of proliferating cells in the DG of prenatally irradiated adult rats. MWR - microwave electromagnetic radiation. Scale bar 100 µm. Mean ± SEM. Statistical significance ** p <0.01, **** p <0.0001.

Cell proliferation was also investigated in the hippocampus of juvenile and adult animals. In irradiated juvenile rats, light microscopic analysis showed a higher density of Ki-67+ cells in the hippocampal DG (Fig. 4A). Quantitative analysis confirmed a significant increase in the number of Ki-67+ cells in the DG of irradiated animals (9.08 ± 0.56 SEM) compared to the number of proliferating cells in control animals (6.38 ± 0.67 SEM, p = 0.0029) (Fig. 4B).

In adult rats exposed to MWR during intrauterine development, light microscopic analysis showed an apparent reduction in cell proliferation in the hippocampal DG compared to control rats (Fig. 4C). Quantitative analysis confirmed a statistically significant decrease in the number of proliferating cells by more than half when compared to the control group (control: 5.369 ± 0.44 SEM, MWR: 2.533 ± 0.30 SEM, p < 0.0001) (Fig. 4D).

#### 3.2.2 Cell death

**Fig. 5:**
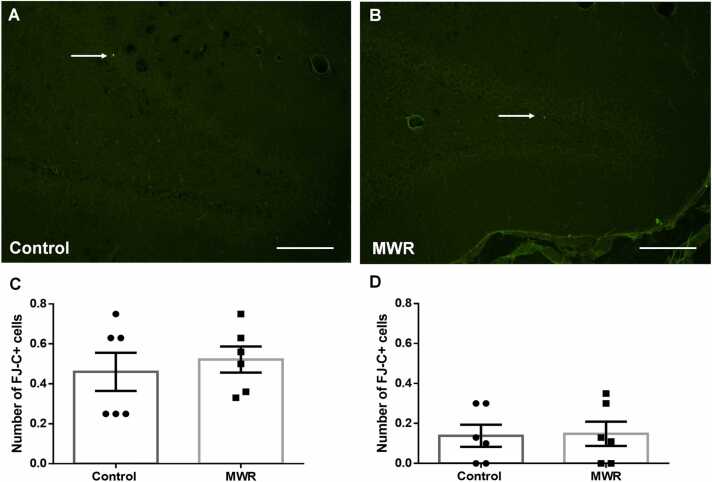
Cell death in the hippocampus of control and prenatally irradiated rats. Representative micrographs showing FJ-C+ cells (arrows) in the DG of the hippocampus of control (A) and prenatally irradiated juvenile (B) rats. The number of dying, FJ-C+ cells in the DG of the hippocampus of control and prenatally irradiated rats. Non-significant differences in cell death between control and prenatally irradiated juvenile (A) (C) and adult (B) (D) rats. Results are expressed as mean ± SEM. Statistical significance ** p <0.01, **** p <0.0001. MWR - microwave electromagnetic radiation.

According to fluorescent microscopic observation, dying, FJ-C+ cells occurred rarely in the DG of the hippocampus in both juvenile control (Fig. 5A) and irradiated (Fig. 5B) rats. Quantitative analysis showed that despite a slight increase in the number of FJ-C+ cells in the DG of irradiated juvenile rats (0.53 ± 0.07 SEM), there was no statistical difference between control and irradiated animals (0.45 ± 0.09 SEM, p = 0.5679) (Fig. 5A C).

Similarly, in adult animals, quantitative analysis showed no significant differences in the number of FJ-C+ cells in the DG on the hippocampus between the control group (0.14 ± 0.04 SEM) and the irradiated group of rats (0.14 ± 0.04 SEM, p = 0.9445) (Fig. 5B D).

### 3.3 Behavior of adult rats after exposure to MWR

#### 3.3.1 Open field test

The results of open field test showed differences only in some of the monitored parameters between the control and irradiated groups (Table 1). Irradiated rats traveled longer distances than control rats and they also moved faster. Rats prenatally exposed to MWR spent significantly more time rearing and reared significantly more often than control rats. These measured parameters between the control and irradiated groups were statistically significant. The remaining parameters such as grooming, time spent in the central zone and the number of entrances to the central zone, the time spent by the walls of open field and defecation were not statistically significant.

**Table 1:** Observed parameters of the control and irradiated group of rats in the open field test. Values are reported as mean ± SEM. Statistical significance: (*) - p <0.05, (**) - p <0.01; ns – insignificant, s – second.

**OPEN FIELD TEST**	**Control group (n = 12)**	**Irradiated group (n = 12)**	**Signification**
Grooming (s)	32,58 ± 6,90	15,46 ± 6,88	ns p = 0,1113
Rearing (number)	**22,50 ± 2,28**	**37,50 ± 3,81**	**p = 0,0094
Apparatus walls (s)	467,40 ± 2,46	470,90 ± 2,75	ns p = 0,3752
Central zone (s)	12,64 ± 2,46	9,11 ± 2,75	ns p = 0,3752
Central zone (frequency)	5,83 ± 0,48	6,38 ± 1,60	
Distance (cm)	**2617,00 ± 186,10**	**3264,00 ± 205,50**	*p = 0,0440
Speed (cm/s)	**5,46 ± 0,39**	**6,82 ± 0,44**	*p = 0,0453
Defecation (number)	5,17 ± 1,05	3,00 ± 0,89	ns p = 0,1388

#### 3.3.2 Elevated plus maze test

The results of behavioral test in the elevated plus maze showed only small differences in the observed parameters between control rats and rats that were exposed to MWR (Table 2). The irradiated rats spent more time in the open arms when compared to the control group. Only this parameter showed statistical significance (Table 2).

**Table 2:** Observed behavior of the control and irradiated rats in the elevated plus maze. Values are expressed as mean ± SEM. Statistical significance: (*) - p <0.05; ns-insignificant, s – second.

**ELEVATED PLUS MAZE TEST**	**Control group (n = 12)**	**Irradiated group (n = 12)**	**Signification**
Rearing (number)	9, 833 ± 2496	10,430 ± 1494	ns p = 0,8361
Groomig (s)	9584 ± 7,07	9889 ± 5,95	ns p = 0,9739
Center crossings (number)	6333 ± 2076	6571 ± 1251	ns p = 0,9209
Open arms (s)	**52,6 ± 15,18**	**119,3 ± 23,34**	*p = 0,0375
Defecation (number)	1833 ± 0,7032	2286 ± 0,6442	ns p = 0,6442

#### 3.3.3 Dark / light box test

Another behavioral test we used to assess the behavior of adult control and irradiated rats was the dark/light box test. Also in this test we observed differences in several monitored parameters (Table 3). Rats exposed to MWR during intrauterine development spent more time in the light part of the box and much less in the dark part of the box and they entered the bright part of the box more often than control rats. Control rats poked their head out for the first time after 5 s, but rats that were exposed to MWR after almost 30 s. These measured parameters were statistically significant.

**Table 3:** Observed parameters of the control and experimental groups of rats in the dark/light box test. Values are expressed as arithmetic mean ± SEM. Statistical significance: (*) - p <0.05, (**) - p <0.01, (***) - p <0.001; ns – insignificant, s – second.

**DARK / LIGHT BOX TEST**	**Control group (n = 12)**	**Irradiated group (n = 12)**	**Signification**
Distance (cm)	**201,80 ± 73,66**	**765,50 ± 130,90**	**p = 0,0021
Speed (cm/s)	**3,95 ± 0,92**	**6,58 ± 0,46**	*p = 0,0196
Light box (s)	**21,76 ± 9,83**	**125,90 ± 21,49**	***p = 0,0006
Dark box (s)	**278,20 ± 9,83**	**174,10 ± 21,49**	***p = 0,0006
Light box (frequency)	**1,00 ± 0,42**	**2,63 ± 0,32**	**p = 0,0086
The number of head pokes	6,88 ± 1,23	5,50 ± 0,78	ns p = 0,3613
First head poke	**5,39 ± 1,52**	**28,87 ± 8,02**	*p = 0,0186
The time spent with head poked	15,03 ± 3,97	8,64 ± 1,71	ns p = 0,1614
Defecation (number)	2,13 ± 0,55	0,75 ± 0,53	ns p = 0,0921

## 4 Discussion

With advances in novel microwave-based systems, MWR has become an integral part of modern life, making it almost impossible to avoid exposure. The developing brain is extremely sensitive to environmental factors including MWR. There are two specific brain regions where neurogenesis continues throughout life, the SVZ of the lateral ventricle and the DG of the hippocampus, however, a substantial part of their development occurs during the embryonic period (Pencea and Luskin, 2003, Rice and Barone, 2000). While radiation-induced changes have been extensively studied in hippocampal neurogenesis and associated animal behavior (Singh et al., 2023; Shahin et al. 2018; Jing et al. 2022; Choi and Choi, 2016; Odaci et al. 2008; Xu et al. 2017), the impact of MWR on the olfactory neurogenic region, the SVZ-RMS-OB, has rarely been investigated (Orendáčová et al., 2009, Orendáčová et al., 2011, Raček et al., 2018).

This study aimed to investigate and compare the effects of MWR on both neurogenic regions within the same animal. We assessed the effect of MWR on neurogenic processes in young and adult animals irradiated during intrauterine development.

Our results showed that the daily two-hour exposure of rats to MWR during the entire period of intrauterine development significantly affected individual processes of postnatal neurogenesis of offspring, depending on the investigated neurogenic region and on the post-irradiation survival time. In addition, the influence of MWR was also manifested by alterations in the behavior of animals that survived into adulthood.

### 4.1 Intrauterine exposure to 2.45 GHz MWR affects the number of proliferating and dying cells in neurogenic regions

In prenatally irradiated rats, we observed that both neurogenic regions respond to the influence of MWR with similar changes in proliferative activity during the postnatal period. In rats surviving to juvenile age, a significant increase in the number of proliferating Ki-67+ cells was observed in both the RMS and the DG. On the contrary, in adulthood, a significant decrease in the number of Ki-67+ cells was noted in both neurogenic regions of irradiated animals. This suggests a biphasic response of the RMS and the DG to the influence of MWR, characterized by an initial up-regulation of cell proliferation followed by its down-regulation after long-term survival.

The observed biphasic pattern aligns with the results reported by Orendáčová et al. (2009), who noted similar changes in proliferating cells number in the RMS of newborn rats exposed to 2.45 GHz MWR. Specifically, short-term exposure induced an increase in BrdU+ cells, while long-term exposure led to a permanent decrease of BrdU+ cells. Reduced cell proliferation in the RMS (Raček et al. 2018) and the DG (Singh et al., 2023) has been observed in rats following postnatal exposure to MWR, suggesting a potential adverse effect of radiation on neurogenesis.

Alterations in proliferation in neurogenic regions either as a result of the influence of external factors or under pathological conditions have been demonstrated in various studies (Schoenfeld and Gould, 2012, Tanapat et al., 2001, Martončíková et al., 2011, Račeková et al., 2009; Bálentová et al., 2006) . Findings across these studies suggest that cell proliferation in neurogenic regions can be enhanced by positive factors, such as learning, exercise, environmental enrichment, and can be decreased by negative factors, such as stress and aging. However, increased cell proliferation and neurogenesis in the neurogenic regions can also by induced by negative stimuli, such as traumatic brain injury (Dash et al. 2001), cerebral ischemia (Jin et al., 2006) and seizure (Parent and Lowenstein, 2002). Reactive increase in adult hippocampal neurogenesis has also been found during abstinence from alcohol dependence (Nawarawong et al. 2021). Another example is a significant increase of proliferating cells number in the RMS of juvenile rats after the paternal exposure to gamma rays (Bálentová et al., 2007). The beneficial effect of reactive neurogenesis after an insult is still a matter of debate. McClain et al. (2014) have found ectopic neuroblasts in the hippocampus of adolescent rats displaying withdrawal symptoms following alcohol dependence, suggesting a potential defect in functional incorporation of newborn cells. In the current study, the MWR induced increase of the number of Ki-67+ cells in juvenile rats was accompanied by an increase of FJ-C+ dying cells. This observation supports a hypothesis that enhanced proliferative activity serves as compensation for cell loss. It can be assumed that this excessive cell proliferation becomes depleted after a certain period, resulting in a subsequent decrease in proliferative activity below control values.

During postnatal production of OB interneurons and hippocampal granule cells, most newborn cells undergo apoptosis to ensure continuous cell turnover under physiological conditions (Biebl et al., 2000, Sierra et al., 2010). In this study, we examined cell death in the RMS and DG of juvenile and adult rats after intrauterine exposition to MWR. Our results revealed that the neurogenic regions responded to radiation by distinct changes in cell death.

In the RMS of both juvenile and adult rats prenatal exposure to MWR caused an increase in the number of dying cells. This increase was more pronounced in young animals, but a highly significant increase in the number of dying cells was still present even in adulthood. Interestingly, in the hippocampus the number of dying cells remained at the level of control values in both juvenile and adult rats. This is consistent with other findings where no Fluoro-Jade+ dying cells were observed in the DG after such a strong intervention as brain inflammation caused by intrahippocampal injection of lipopolysacharide from *Salmonella enterica* (Jakubs et al. 2008).

The observed morphological differences in the response of the RMS and the DG to the effect of MWR support the finding that there are fundamental differences between SVZ and SGZ neurogenesis, whether it concerns to the differential neurogenic potential, regulatory mechanisms or niche-derived extracellular factors (Ertaylan et al. 2014).

### 4.2 Intrauterine exposure to 2.45 GHz MWR attenuates the maturation of nitrergic neurons in the RMS

Based on our previous findings regarding the presence of NO-producing mature neurons in the RMS (Račeková et al. 2005), we investigated the effect of MWR on nitrergic cells in the RMS. Morphological analysis revealed that MWR attenuates the maturation of nitrergic neurons in the RMS of prenatally irradiated rats. NO-producing neurons retained immature neuronal features in juvenile age and even in adulthood. In addition their number was significantly lower compared to age-matched control animals. In contrast to these results, a previous experiment from our laboratory demonstrated that a stressful experience – maternal separation during the early postnatal period – caused premature differentiation of NO-producing cell in the RMS in addition to altered cell proliferation and death (Lievajová et al. 2011). This suggests that the maturation of nitrergic cells in the RMS can be influenced by various external factors, depending on the nature of the factors and the period of life when they are applied. Based on the important regulatory role of NO in postnatal neurogenesis (Packer et al., 2003, Moreno-López et al., 2004), the observed alterations in nitrergic cells maturation indicate the contribution of NO on altered proliferation and cell dying induced by external factors.

As mature nitrergic neurons of the RMS have been found to form synaptic connections and these neurons are probably involved in an existing neuronal circuit (Blaško et al. 2013), we assume that developmental failure of nitrergic neurons may lead to permanent dysfunction of their neuronal network in adulthood.

### 4.3 MWR induces changes in animals’ behavior

Morphological changes in neurogenesis observed in adult rats exposed to MWR during intrauterine development were accompanied by obvious changes in animals’ behavior. These findings are generally consistent with the results of other studies that have shown that prenatal exposure to MWR can produce different effects on animals’ behavioral tasks (Galvin et al., 1986, Aldad et al., 2012, Zhang et al., 2015). In our study, open-field testing of locomotor activity showed a significant increase in distance traveled and average speed of prenatally irradiated animals inside the testing apparatus. We observed the same result in the dark/light box test, where besides the increased distance and speed, a significant increase in the number of entrances into the light compartment was also detected. Our findings are in agreement with the study of Aldad et al. (2012), according to which the enhancement in locomotor activity and a higher number of transitions between two compartments of dark/light box in the irradiated group suggest hyperactive behavior. In addition to locomotor hyperactivity, Aldad et al. (2012) found decreased memory and decreased anxiety in prenatally irradiated animals. Similarly, in our study, prenatal exposure to MWR caused decrease in several anxiety-related behavioral parameters assessed in the open-field, elevated plus maze and dark/light box tests.

Changes in behavioral functions are mostly attributed to alterations in neurogenesis in the hippocampus (Ganapathi and Manda, 2017), which may be related to the fact that higher brain functions are thought to be maintained by hippocampal neurogenesis. However, our results showed distinct alterations in the processes of neurogenesis also in the RMS, suggesting that the observed behavioral abnormalities could also be related to changes in neurogenesis in the migratory pathway. Newly generated OB interneurons are necessary for preserving normal odor information processing (Malvaut and Saghatelyan, 2016). Indeed, in another experimental paradigm, following cranial irradiation of adult mice by gamma rays, marked decrease of new OB neurons was found accompanied by impairment of long-term olfactory memory (Lazarini et al., 2009).

The excessive use of smartphones combining various media functions in one device is currently often associated with an increasing incidence of attention-deficit- hyperactivity disorder (ADHD) in both children and adults (Ko et al., 2008, Panagiotidi, 2017; (Panagiotidi and Overton, 2022)). Karsz et al. (2008) when examining the olfactory abilities of children with ADHD, reported significant olfactory deficits in these children. Impaired olfactory processing has been observed also in adults with ADHD (Brewer et al. 2003). Similarly to human ADHD patients, olfactory dysfunction has been found in ADHD mice model (Sim et al. 2023). The authors assume that the olfactory impairment in ADHD mice is related to the decreased differentiation of the SVZ neural stem cells. We suppose that the hyperactive behavior observed in this study following MWR exposure may also be associated with impaired olfaction as a consequence of changes in cell proliferation and cell dying in the RMS.

## 5 Conclusion

The postnatal generation of new neurons in the SVZ and the SGZ is crucial for the normal brain function. MWR appears to significantly affect neuronal turnover in these neurogenic regions. The present results revealed that chronic intrauterine exposure to 2.45 GHz MWR induced biphasic changes in cell proliferation in the RMS and the DG. Specifically, the results demonstrate a remarkable increase in the number of proliferating cells in the offspring that survived to juvenile age, followed by a marked reduction in cell proliferation in adulthood. In the RMS, increased cell death and impaired maturation of nitrergic neurons were also observed in both juvenile and adult animals. Moreover, the long-term effect of MWR was reflected in neurobehavioral changes, including locomotor hyperactivity in adult animals. Given the prevalence of constant MWR exposure in our daily lives, complete elimination of such exposure is practically impossible. However, the experimental models stimulating realistic human exposure are still not consistent and comparable. Extrapolation of experimental results to human requires further studies.

## CRediT authorship contribution statement

**Monika Žideková:** Writing – review & editing, Conceptualization. **Adam Raček:** Methodology. **Kamila Fabianová:** Visualization, Data curation. **Marcela Martončíková:** Project administration, Funding acquisition, Formal analysis. **Enikő Račeková:** Supervision, Investigation. **Alexandra Popovičová:** Writing – original draft.

## Declaration of Competing Interest

The authors declare that they have no known competing financial interests or personal relationships that could have appeared to influence the work reported in this paper. The authors have read and have abided by the statement of ethical standards for manuscripts submitted to IBRO Neuroscience Reports.
